# Supplementary material for: Journal research data policies in materials science
Source: Digit Discov. 2026 Jun 8;5(7):2795–808. doi: 10.1039/d6dd00111d (PMC13266401; doi:10.1039/d6dd00111d)
Supplement: DD-005-D6DD00111D-s001 [file DD-005-D6DD00111D-s001.pdf]

Supplementary Information (SI) for Digital Discovery.
This journal is © The Royal Society of Chemistry 2026

Supplementary Information: Journal Research Data Policies in Materials Science

Lukas Hörmann,[1,2] Hemanadhan Myneni,[3] Rwayda Kh. S. Al-Hamd,[4] Katarina Batalović,[5] Silvia Bonfanti,[6] Federico Grasselli,[7,8] Saulius Gražulis,[9] Bahattin Koç,[10] Konstantinos Konstantinou,[11] Ivor Lončarić,[12] Nataliya Lopanitsyna,[13] José Manuel Oliveira,[14,15] Paolo Pegolo,[16] Patrícia Ramos,[14,17] Kevin Rossi,[18,19] Sebastian P. Schwaminger,[20,21] Edith Simmen,[22] Milica Todorović,[11] Markus Stricker,[23,*] and Jonathan Schmidt[22,16]

[1]*Faculty of Physics, University of Vienna, Vienna, 1090, Austria*
[2]*Department of Chemistry, University of Warwick, Coventry, CV4 7AL, United Kingdom*
[3]*The Faculty of Industrial Engineering, Mechanical Engineering, and Computer Science, University of Iceland, Reykjavík, Iceland*
[4]*Department of Civil Engineering and Management, Faculty of Science and Engineering, The University of Manchester, Manchester, M13 9PL, United Kingdom*
[5]*Vinča Institute of nuclear sciences-national institute of the Republic of Serbia, University of Belgrade, Serbia*
[6]*NOMATEN Centre of Excellence, National Center for Nuclear Research, ul. A. Sołtana 7, 05-400 Swierk/Otwock, Poland.*
[7]*Dipartimento di Scienze Fisiche, Informatiche e Matematiche, Università degli Studi di Modena e Reggio Emilia, 41125 Modena, Italy*
[8]*CNR-NANO S3, 41125 Modena, Italy*
[9]*Vilnius University, Life Sciences Center, Institute of Biotechnology, Saulėtekio al. 7, LT-10257 Vilnius, Lithuania*
[10]*Sabanci University, Orta Mah, FENS 1023, Tuzla, Istanbul, Turkey*
[11]*Department of Mechanical and Materials Engineering, University of Turku, Turku 20014, Finland*
[12]*Ruđer Bošković Institute, HR-10000 Zagreb, Croatia*
[13]*Syngenta Crop Protection AG, Schaffhauserstrasse, Stein, 4332, AG, Switzerland*
[14]*Institute for Systems and Computer Engineering, Technology and Science, 4200-465 Porto, Portugal*
[15]*School of Economics and Management, University of Porto, 4200-464 Porto, Portugal*
[16]*Laboratory of Computational Science and Modeling, Institut des Matériaux, École Polytechnique Fédérale de Lausanne, 1015 Lausanne, Switzerland*
[17]*CEOS.PP, ISCAP, Polytechnic of Porto, 4465-004 S. Mamede de Infesta, Portugal*
[18]*Department of Materials Science and Engineering, Delft University of Technology, Delft, Netherlands*
[19]*Climate Safety and Security Centre, TU Delft The Hague Campus, Delft University of Technology, 2594 AC, The Hague, The Netherlands*
[20]*NanoLab, Division of Medicinal Chemistry, Otto Loewi Research Center, Medical University of Graz, Neue Stiftingtalstraße 6, 8010, Graz, Austria*
[21]*BioTechMed-Graz, Mozartgasse 12, 8010 Graz, Austria*
[22]*Department of Materials, ETH Zürich, Zürich, CH-8093, Switzerland*
[23]*Interdisciplinary Centre for Advanced Materials Simulation, Ruhr University Bochum, D-44799 Bochum, Germany*
(Dated: May 15, 2026)

A. Reviewing consistency

As we mentioned in the Methods in the main manuscript, each RDP was encoded by at least two persons. This allows us to study the consistency of encodings, which is shown in Fig. 1. The comparison reveals substantial inconsistencies in the interpretation of open data policies across publishers. For nearly all major publishers, some journals are coded as consistent, while others exhibit cases of missed text or comprehension issues. This indicates that even when policies exist, their wording and presentation are often ambiguous or difficult to interpret consistently. The presence of both missed text and comprehension categories suggests that the clarity, accessibility, and standardization of policies remain uneven, which may contribute to confusion among authors and editors about what is required in practice.

* Corresponding author: markus.stricker@rub.de

B. Statistical tests

Pearson correlation coefficient and significance test. The Pearson correlation coefficient r quantifies the strength and direction of the linear relationship between two variables x and y:

$$r = \frac{\sum_{i=1}^{n}(x_i - \bar{x})(y_i - \bar{y})}{\sqrt{\sum_{i=1}^{n}(x_i - \bar{x})^2}\sqrt{\sum_{i=1}^{n}(y_i - \bar{y})^2}} \quad (1)$$

Here $\bar{x}$ and $\bar{y}$ denote the sample means of x and y, respectively. n is the number of paired observations. The value of r ranges from -1 (perfect negative correlation) to $+1$ (perfect positive correlation), with $r = 0$ indicating no linear correlation. To test whether the observed correlation is statistically significant, the following two hypotheses are formulated:

$$H_0 : \rho = 0 \quad \text{vs.} \quad H_1 : \rho \neq 0$$

Here ρ is the (unknown) population correlation. Under the null hypothesis H_0, the test statistic follows a Stu-

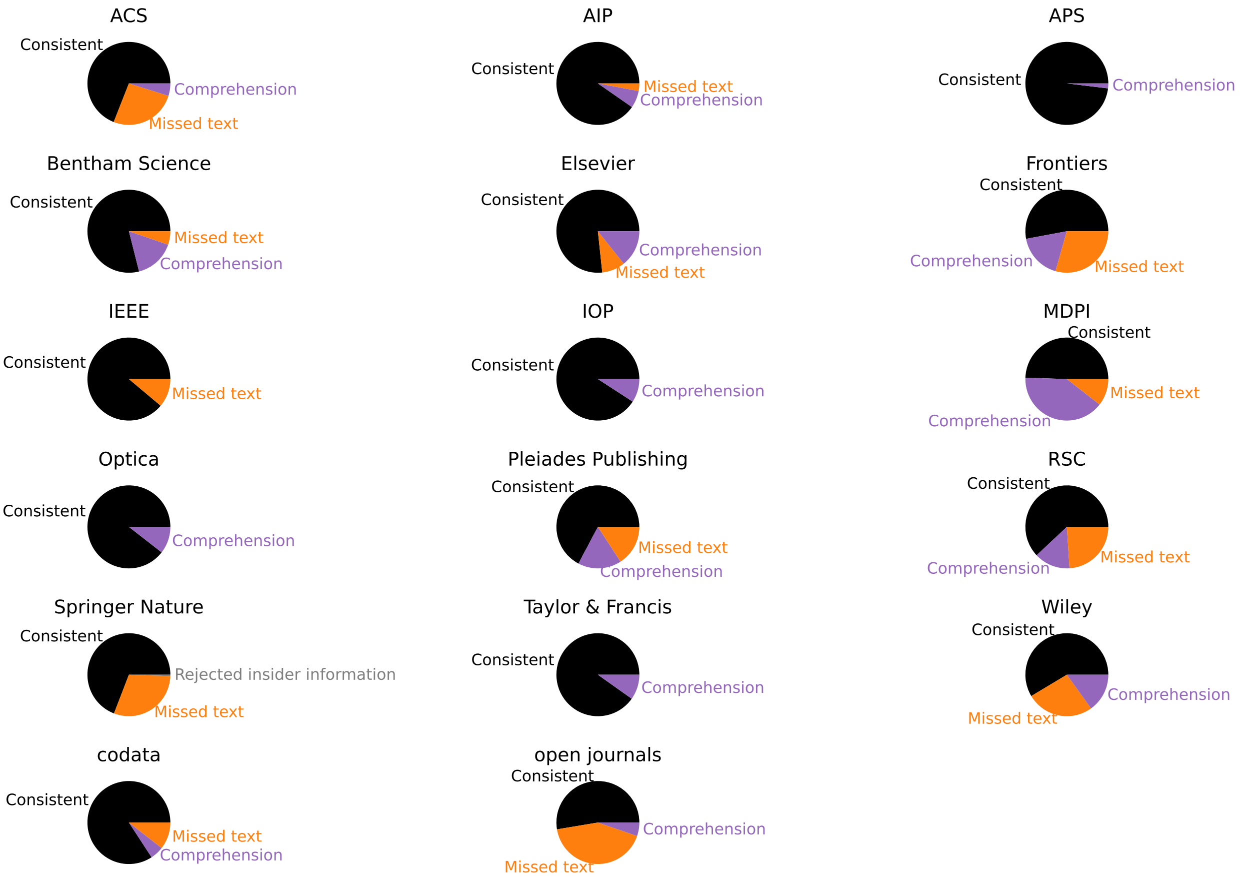

FIG. 1. Inconsistencies encountered during the encoding process for different publishers.

dent's t-distribution with $\nu = n - 2$ degrees of freedom.

$$t = \frac{r}{\sqrt{1 - r^2}} \sqrt{n - 2}$$

The p-value is computed in the following equation, where $T_{(\nu)}$ follows a Student's t-distribution with ν degrees of freedom.

$$p = 2\, P\left(T_{(\nu)} > |t|\right)$$

The p-value is the probability of observing a test statistic that is at least as extreme as what is actually observed, under the assumption that the null hypothesis H_0 is true. A small p-value indicates that the observed correlation r is unlikely to have arisen under the null hypothesis. Therefore, we can reject H_0 in favor of H_1.

One-Way ANOVA. The one-way analysis of variance (ANOVA) is used to test whether the means of k independent groups are significantly different. It compares the variance between groups to the variance within groups. The null hypothesis it that the true means μ_i are equal:

$$H_0 : \mu_1 = \mu_2 = \cdots = \mu_k$$

Let X_{ij} denote the j-th observation in group i, with n_i observations per group ($i = 1, \ldots, k$), and $N = \sum_{i=1}^{k} n_i$ total observations. The observed mean $\bar{X}_i$ of group i is given by the following equation:

$$\bar{X}_i = \frac{1}{n_i} \sum_{j=1}^{n_i} X_{ij}$$

The overall mean is $\bar{X}$. The test statistic is the ratio of these mean squares of the mean-square-between-groups (MSW) mean-square-within-groups (MSW).

$$F = \frac{\text{MSB}}{\text{MSW}}$$

MSB quantifies the variability between groups:

$$\text{MSB} = \frac{1}{k-1} \sum_{i=1}^{k} n_i (\bar{X}_i - \bar{X})^2,$$

MSW measures the variability within each group:

$$\text{MSW} = \frac{1}{N-k} \sum_{i=1}^{k} \sum_{j=1}^{n_i} (X_{ij} - \bar{X}_i)^2,$$

F follows an F-distribution with $(k-1,\, N-k)$ degrees of freedom. The corresponding p-value is given by

$$p = P\big(F_{(k-1,\,N-k)} > F_{\text{obs}}\big).$$

C. Data acquisition

List of whether a journal is "open access" or not was generated with generative AI (ChatGPT GPT-5).

D. Examplary result for open data score assignment

All full YAML files can be found at Github.

Listing 1. Springer Nature Research Data Policy (RDP) Assessment


```
rdp_exist:
  text: 1. Existence of research data policy
  N. encoders: 2
  has_discrepancies: false
  0:
    text: Research Data Policy (RDP) exists.
    explanation: 'At Springer Nature we advance
        discovery by publishing trusted research,
      supporting the

      development of new ideas and championing open
          science. We also aim to facilitate

      compliance with research funder and institution
          requirements to share data.


      To help accomplish this we have established a
          standard research data policy
      for our

      journals, based on transparency around supporting
           data. This policy applies
      to all datasets

      that are necessary to interpret and replicate the
           conclusions reported in a
      research article.'
  1:
    text: Research Data Policy (RDP) exists.
    explanation: This journal follows Springer Nature
        research data policy.
  correct_answer: null
  discrepancy_reason: null

data_sharing:
  text: 3. Data sharing requirements in RDP
  N. encoders: 2
  has_discrepancies: true
  0:
    text: Public data sharing required only for
        specific types of data.
    explanation: 'We encourage authors to deposit their
         supporting data in publicly
      available repositories, or

      failing this within the manuscript or additional
          supporting files. See our repository
      guidance

      for more information.


      For a number of data types, submission to a
          community-endorsed, public repository
      is

      mandatory. See our list of mandated data types.'
  1:
    text: Data sharing encouraged but optional.
    explanation: Sharing of all relevant research data
        is strongly encouraged
  correct_answer:
    text: Public data sharing required only for
        specific types of data.
    explanation: 'We encourage authors to deposit their
         supporting data in publicly
      available repositories, or

      failing this within the manuscript or additional
          supporting files. See our repository
      guidance

      for more information.


      For a number of data types, submission to a
          community-endorsed, public repository
      is

      mandatory. See our list of mandated data types.'
  discrepancy_reason: Text not found

data_fair:
  text: 4. FAIR data sharing (see https://www.go-fair.
      org/fair-principles/ for a definition
    of FAIR)
  N. encoders: 2
  has_discrepancies: true
  0:
    text: Public data sharing on a FAIR repository
        required only for specific types
      of data (e.g. genetic data has to be shared on a
          FAIR repository but no other
      data).
    explanation: "For the following data types
        submission to a community-endorsed,\
      \ public repository is\nmandatory. Persistent
          identifiers (DOIs and accession\
      \ numbers) assigned to the data by the\
          nrepository must be appropriately cited\
      \ and referenced in the published article.\n\
          nCrystallographic data for small\
      \ molecules \u2192 Cambridge Structural Database
          (CSD/CCDC)\n\n[Note: CCDC supports\
      \ FAIR Data principles: see:\nhttps://www.ccdc.
          cam.ac.uk/solutions/about-the-csd/fair-data-
          principles/\n\
      https://www.ccdc.cam.ac.uk/discover/blog/our-
          approach-to-the-fair-data-principles/]"
  1:
    text: Public data sharing on a FAIR repository
        encouraged.
    explanation: Authors are strongly encouraged to
        deposit their supporting data
      in a publicly available repository. Sharing your
          data in a repository promotes
      the integrity, discovery and reuse of your
          research, making it easier for the
      research community to build on and credit your
          work.
  correct_answer:
    text: Public data sharing on a FAIR repository
        required only for specific types
      of data (e.g. genetic data has to be shared on a
          FAIR repository but no other
      data).
    explanation: "For the following data types
        submission to a community-endorsed,\
      \ public repository is\nmandatory. Persistent
          identifiers (DOIs and accession\
      \ numbers) assigned to the data by the\
          nrepository must be appropriately cited\
      \ and referenced in the published article.\n\
          nCrystallographic data for small\
      \ molecules \u2192 Cambridge Structural Database
          (CSD/CCDC)\n\n[Note: CCDC supports\
      \ FAIR Data principles: see:\nhttps://www.ccdc.
          cam.ac.uk/solutions/about-the-csd/fair-data-
          principles/\n\
      https://www.ccdc.cam.ac.uk/discover/blog/our-
          approach-to-the-fair-data-principles/]"
  discrepancy_reason: Text not found

data_availability:
  text: 2. Data availability statement
  N. encoders: 2
  has_discrepancies: false
  0:
    text: Required according to the RDP.
    explanation: 1. All original articles must include
        a data availability statement
  1:
    text: Required according to the RDP.
    explanation: 'All original research must include a
        data availability statement. '
  correct_answer: null
  discrepancy_reason: null
```
